# Immune checkpoint inhibitor PD-1 pathway is down-regulated in synovium at various stages of rheumatoid arthritis disease progression

**DOI:** 10.1371/journal.pone.0192704

**Published:** 2018-02-28

**Authors:** Yanxia Guo, Alice M. Walsh, Mary Canavan, Mihir D. Wechalekar, Suzanne Cole, Xuefeng Yin, Brittney Scott, Mathew Loza, Carl Orr, Trudy McGarry, Michele Bombardieri, Frances Humby, Susanna M. Proudman, Costantino Pitzalis, Malcolm D. Smith, Joshua R. Friedman, Ian Anderson, Loui Madakamutil, Douglas J. Veale, Ursula Fearon, Sunil Nagpal

**Affiliations:** 1 Immunology, Janssen Research, Pennsylvania, United States of America; 2 Molecular Rheumatology, Trinity Biomedical Sciences Institute, Trinity College, Dublin, Ireland; 3 Rheumatology Unit, Repatriation General Hospital and Flinders University, Adelaide, Australia; 4 Centre for Arthritis and Rheumatic Diseases, St. Vincent's University Hospital, University College Dublin, Elm Park, Dublin, Ireland; 5 Queen Mary University of London, Charterhouse Square, London, United Kingdom; 6 Rheumatology Unit, Royal Adelaide Hospital, Adelaide, Australia and Discipline of Medicine, University of Adelaide, Adelaide, Australia; Universite de Nantes, FRANCE

## Abstract

Immune checkpoint blockade with therapeutic anti-cytotoxic T lymphocyte-associated antigen (CTLA)-4 (Ipilimumab) and anti-programmed death (PD)-1 (Nivolumab and Pembrolizumab) antibodies alone or in combination has shown remarkable efficacy in multiple cancer types, concomitant with immune-related adverse events, including arthralgia and inflammatory arthritis (IA) in some patients. Herein, using Nivolumab (anti-PD-1 antagonist)-responsive genes along with transcriptomics of synovial tissue from multiple stages of rheumatoid arthritis (RA) disease progression, we have interrogated the activity status of PD-1 pathway during RA development. We demonstrate that the expression of *PD-1* was increased in early and established RA synovial tissue compared to normal and OA synovium, whereas that of its ligands, programmed death ligand-1 (*PD-L1*) and *PD-L2*, was increased at all the stages of RA disease progression, namely arthralgia, IA/undifferentiated arthritis, early RA and established RA. Further, we show that RA patients expressed PD-1 on a majority of synovial tissue infiltrating CD4^+^ and CD8^+^ T cells. Moreover, enrichment of Nivolumab gene signature was observed in IA and RA, indicating that the PD-1 pathway was downregulated during RA disease progression. Furthermore, serum soluble (s) PD-1 levels were increased in autoantibody positive early RA patients. Interestingly, most of the early RA synovium tissue sections showed negative PD-L1 staining by immunohistochemistry. Therefore, downregulation in PD-1 inhibitory signaling in RA could be attributed to increased serum sPD-1 and decreased synovial tissue PD-L1 levels. Taken together, these data suggest that agonistic PD1 antibody-based therapeutics may show efficacy in RA treatment and interception.

## Introduction

Immune check-point blockade has shown unprecedented superiority to prior therapies in cancer treatment as demonstrated by significantly prolonged survival of patients given anti-cytotoxic T lymphocyte-associated antigen-4 (anti-CTLA-4, Ipilumimab) [1], anti-programmed death-1 (anti-PD-1, Pembrolizumab and Nivolumab) [2, 3], or anti-PD-L1 (anti-PD-Ligand 1, Atezolizumab) [4] antibodies. Immune modulatory activities of these novel drugs are essential for the robust clinical efficacy observed in cancer patients. Given that these co-inhibitory molecules are part of the immune homeostatic mechanisms that dampen overt immune activation [5, 6], it is natural to predict immunotherapy to be a double-edged sword that activates immune cells to attack cancer cells but also harbors the potential to unleash self-antigen reactive T cells, thus leading to inflammatory and autoimmune diseases. In fact, studies have revealed immune-related adverse events (irAE) in a subset of patients treated with Ipilimumab, Nivolumab, or Pembrolizumab or a combination of anti-CTLA-4 and anti-PD-1 therapeutics. The checkpoint blockade adverse events include arthralgia [7], inflammatory arthritis (IA) [8], tenosynovitis [9], colitis [10] and psoriasis [11]. A comparative study of checkpoint blockade-mediated IA and the naturally occurring cases of the related disease during rheumatoid arthritis (RA) evolution may provide insight into the mechanism(s) of disease development and progression and uncover therapeutic pathways for the treatment and prevention.

Disease development in RA starts with asymptomatic autoimmunity in susceptible subjects, which transitions to arthralgia stage as autoantibodies gain access to joints and patients start experiencing non-specific musculoskeletal symptoms without any clinical signs of synovitis. Over time, arthralgia patients progress to IA/undifferentiated arthritis (UA) when they develop clinical synovitis, and finally with increased immune cell infiltration and epitope spreading, IA/UA patients develop clinically classifiable RA [12, 13]. Joint manifestation in RA results from synovial hyperproliferation and infiltration of immune cells into the synovium leading to bone resorption and cartilage degradation [13]. Presence of T cells as a major component of the synovial tissue immune cell infiltrate [14, 15], identification of T cell activation and function genes as risk alleles in genome-wide association studies and clinical success of T cell-based therapeutics (e.g., Abatacept) [15, 16] have implicated T cells as a major player in the pathogenesis of RA. Considering the involvement of activated T cells and the development of arthralgia, synovitis, tenosynovitis and IA in some of the cancer patients receiving anti-PD-1 antibody therapeutics, we hypothesized that dysregulated PD1-PD-L1/PD-L2 pathway may be involved in the progression of arthralgia and IA/UA to early and established RA. Herein, to characterize molecular underpinnings of disease progression, we have examined PD-1 pathway at various stages of RA development by interrogating the enrichment of Nivolumab-induced human tumor microenvironment (TME) gene signature in arthralgia, IA/UA, early RA and established RA. We demonstrate that the activity of PD-1 pathway was down-regulated at various stages of RA disease progression despite high PD-1 expression on RA synovial tissue infiltrating CD4^+^ and CD8^+^ T cells. Moreover, anti-PD-1 TME gene signature was reduced after tDMARD treatment of early RA patients. In addition, we show that soluble PD-1 (sPD-1) levels were increased in serum samples of ACPA-positive but not ACPA-negative RA subjects. We also demonstrate that most of the early RA synovial tissue samples did not exhibit PD-L1 protein staining by immunohistochemistry, whereas CD3 and PD-1 proteins were readily detected in these tissue sections. These results not only indicate the involvement of the PD-1 pathway in disease pathogenesis and progression but also underscore the importance of targeting PD-1 pathway both for RA treatment and interception.

## Materials and methods

### Human subjects

Human donors (healthy, Osteoarthritis [OA], arthralgia, UA, early RA and established RA) used in this study have been described previously [17]. Briefly, synovial biopsy specimens for healthy and OA donors were obtained from a group of patients attending a sports medicine facility with knee pain. Healthy subjects were defined as those who had no evidence of any form of arthritis and had no cartilage damage or synovitis on knee arthroscopy. OA donors had a clinical history and/or examination findings suggestive of OA in addition to supportive arthroscopic findings. The average age of healthy donors was 35.2 years (range 20–66 years). There were 14 females (50%) and 14 males (50%). The average age of OA donors was 49.0 years (range 19–69 years). There were 13 females (59.1%) and 9 males (40.9%). Arthralgia patients (n = 10) were defined as subjects with symptoms of aches and pains, without clinical signs of synovitis or significantly raised C-reactive protein (CRP) (mean = 4.49 mg/l) at first assessment, but with positive circulating rheumatoid factor (RF^+^) and anti-citrullinated protein antibodies (ACPA). There were eight females and two males, and the mean age was 51.6 years (range 34–66 years). Undifferentiated arthritis/inflammatory arthritis (UA/IA) patients (n = 6) were defined as subjects presenting with clinical signs of synovitis, but who failed to meet the 2010 ACR criteria for RA. All 6 patients were females, and the mean age was 46 years with significantly raised CRP (mean = 17.66mg/l). All patients were RF negative and 2 were ACPA positive at the time of sample collection. Early RA (n = 57) was defined as within 12 months of disease diagnosis without prior small or large molecule disease modifying anti-rheumatic drugs (DMARDs) usage. Early RA synovial tissue biopsies were collected from 2 different cohorts of patients identified at Flinders University and Queen Mary University of London. There were 33 females and 22 males, and the mean age was 55.9 years (range 25–89 years) at the time of the sample collection. The mean disease duration was 5 months. Established RA (n = 95; more than 1 year of disease duration) synovial tissue biopsies were collected from two different cohorts of patients identified at Queen Mary University of London and St. Vincent’s Hospital. The average disease duration in this patient population was 68 months. The average age for the group was 54.0 years (range 24–85 years). All established RA patients had received small molecule DMARDs or anti-TNFa treatments.

Flinders university early RA synovial biopsies (at baseline and at 6 months follow-up) were done as a part of a research study, fully approved by the Southern Adelaide Local Health Network Human Research Ethics Committee. This protocol is freely available on the ANZ trial network (ANZCTR; protocol details ACTRN12611001202954). Queen Mary University of London early RA synovial tissue biopsies were obtained as a part of the Pathobiology of Early Arthritis Cohort (PEAC) study (05/Q0703/198). The clinical protocol is available publicly (http://www.peacmrc.mds.qmul.ac.uk/docs/PEAC%20PROTOCOL-Protected.pdf), and the protocol was approved by the Kings College Hospital Ethics Committee. Subjects with arthralgia, but no clinical synovitis, who had positive RF and ACPA were recruited from rheumatology clinics at St. Vincent’s University Hospital according to the research protocol: ‘Identification of Genes and Cytokine Profiles Associated with an Enhanced Inflammatory Response and Prediction of Therapeutic Response’ approved by the St. Vincent’s Healthcare Group Medical Research and Ethics Committee. Synovial biopsy was performed by needle arthroscopy under local anesthetic following fully-informed, written consent.

For the analysis of sPD-1, healthy control serum samples were received from Bioreclamation (West Bury, NY), Janssen R&D healthy donors (n = 20), and Flinders University (n = 33). Early RA serum samples were collected at Flinders University. All protocols for collecting synovial biopsies and blood/serum were approved by Institutional Review Board. All patients signed the consent form for participating in the study.

All protocols for collecting synovial biopsies and blood were approved by the Southern Adelaide Local Health Network Human Research Ethics Committee, Kings College Hospital Ethics Committee or St. Vincent’s Healthcare Group Medical Research and Ethics Committee. All patients gave written informed consent for participation in the study.

### Flow cytometry analysis

Samples used for synovial-infiltrating T cell analysis were collected at The Centre for Arthritis and Rheumatic Diseases, St. Vincent’s University Hospital, UCD. Synovial tissue biopsies were digested using a soft tumor dissociation kit (Miltenyi Biotech) per manufacturer’s instructions. The following antibodies were used in FACS analysis: CD45-PerCPCy5.5, CD3-FITC, CD4-PE, CD8-PECy5, CD45RO-PECy7, PD1-Brilliant violet 421, and Live/Dead-Aqua (or Live/Dead Red). All Abs were purchased from BD Bioscience. Fluorescene Minus One (FMO) controls were used to determine gating boundaries. Samples were acquired using the CyAn Flow Cytometer (Beckman Coulter) and analyzed using Flowjo software (Treestar Inc.).

### T cell proliferation

CD4^+^ Memory T cells were isolated from PBMC of healthy and RA donors using StemCell Technology kit. Healthy PBMC were ordered from Biological Specialty (Colmar, PA) and RA PBMC were from Bioreclamation. CD4^+^ memory cells were stimulated with anti-CD3 (1μg/ml, eBioscience) in the presence of human IgG1 or cyno PD-L1-Fc (1μg/ml) for 72hr in 96-well plates. ^3^H (5μCi) was added to each well and incubated for 6hr before the read-out. Both anti-CD3 antibodies and cyno-PD-L1-Fc (or human IgG1 control antibodies) were used in a plate-bound format. Expression vector encoding the extracellular domain (ECD) of cynomolgus PD-L1 as Ig fusion protein was prepared, with the ECD attached via a short linker to the hinge-CH2-CH3 domains of human IgG1. The plasmid DNA was transfected into the Expi293 expression system (Thermo- Fisher, Waltham, MA) and supernatant was harvested after 5 days. The protein was purified directly from cell culture supernatants by protein A Sepharose, and then dialyzed into phosphate buffered saline (PBS), pH 7.2. Protein concentration was measured by absorbance at 280 nm, and HPLC as well as SDS-PAGE analysis were used to confirm purity.

### MSD assay for sPD-1

The sPD-1 assay is a customized assay developed on the Meso Scale Discovery (MSD) platform using the capture and detection antibodies from R&D Systems’ Human PD-1 DuoSet (DY1086). The assay has a sensitivity of 6.4 pg/mL, passing qualification standards of 95–123% spike-recovery, 0.89–18.7% intra-assay and 10–14% inter-assay variability in serum. Serum samples were measured at an 8-fold dilution with concentration calculated based on a standard curve.

### PD-L1 immunohistochemistry and analysis

Formalin-fixed paraffin-embedded early RA synovial biopsies were cut into 5μM sections and mounted on Fisherbrand Superfrost Plus Slides. The sections were stained with anti-CD3 (Cat# 103R-96, Cell Marque) and anti-PD1 (Cat# ab137132, Abcam) antibodies. PD-L1 analysis was performed using PD-L1 IHC 22C3 pharmDx following the manufacture instructions. Human tonsil and cell lines expressing PD-L1 (IHC 22C3 pharmDx control cell line slide) were used as positive controls for PD-L1 staining. The markers were visualized with 3,3'-diaminobenzidine and the relative expression of each marker was noted. CD3 and PD1 staining was reported as CD3^+^% or PD1^+^% cells within a total magnification of 100x across the entire biopsy section. PD-L1 staining was denoted as <1%, ≥1% or ≥50%, respectively.

### RNA-Seq gene expression analysis

RNA-Seq analysis was performed on synovial tissue biopsies using the algorithm reported previously [17]. Briefly, total RNA was extracted from synovial tissue biopsies and the quality was evaluated using an Agilent Bioanalyzer. Sequencing libraries were prepared using TruSeq Stranded Total RNA RiboZero protocol from Illumina. Libraries were pooled and sequenced with an Illumina HiSeq 2000 with paired-end 100 bp flow cells. Raw read quality was evaluated using FastQC. Reads were trimmed for adaptors and sequence quality. The average number of clusters (post-trimming) per sample was 8.9x10^7^. Trimmed reads were aligned to human b37.3 reference genome using the STAR v2.4 aligner. Aligned reads were quantified using RSEM v1.2.14 algorithm with UCSC transcriptome model (accessed on 03/17/2014) that included lincRNAs from Ensembl v75. Aligned data was evaluated for quality using several metrics (e.g., mapping rate, coverage) and visually inspected for deviation from the population across multiple metrics and principal components analysis. Statistical testing of RNA-seq data was performed in R with the “limma” package. Counts were converted to log2 counts per million, quantile normalized and precision weighted. A linear model was fitted to each gene, and empirical Bayes moderated t-statistics were used to assess differences in expression. The RNA-Seq data was deposited in GEO database (GSE89408) [17].

### Gene set variation analysis (GSVA)

GSVA was performed in R using the “GSVA” package as described previously [18]. The signature set of Nivolumab-induced genes was identified from a human metastatic renal carcinoma study [19]. The enrichment scores were calculated using the magnitude difference between the largest positive and negative random walk deviations. Two-sided T-tests were used to compare enrichment scores between groups.

### Statistics

For high-dimensional RNA-seq, features were considered differentially expressed if they satisfied a 1.5 fold-change and 0.05 adjusted p-value cutoff unless otherwise specified. The Benjamini-Hochberg method was used to calculate p-values adjusted for multiple hypotheses. Fold-changes were calculated from log2(fold-change) estimates and reported with a positive sign for ratios greater than 1 (log2(fold-change) > 0) and with a negative sign for ratios less than 1 (log2(fold-change) < 0).

## Results

### *PDCD1*, *CD274* and *PDCD1LG2* expression is up-regulated in synovial tissue at various stages of RA disease progression

**Fig 1:**
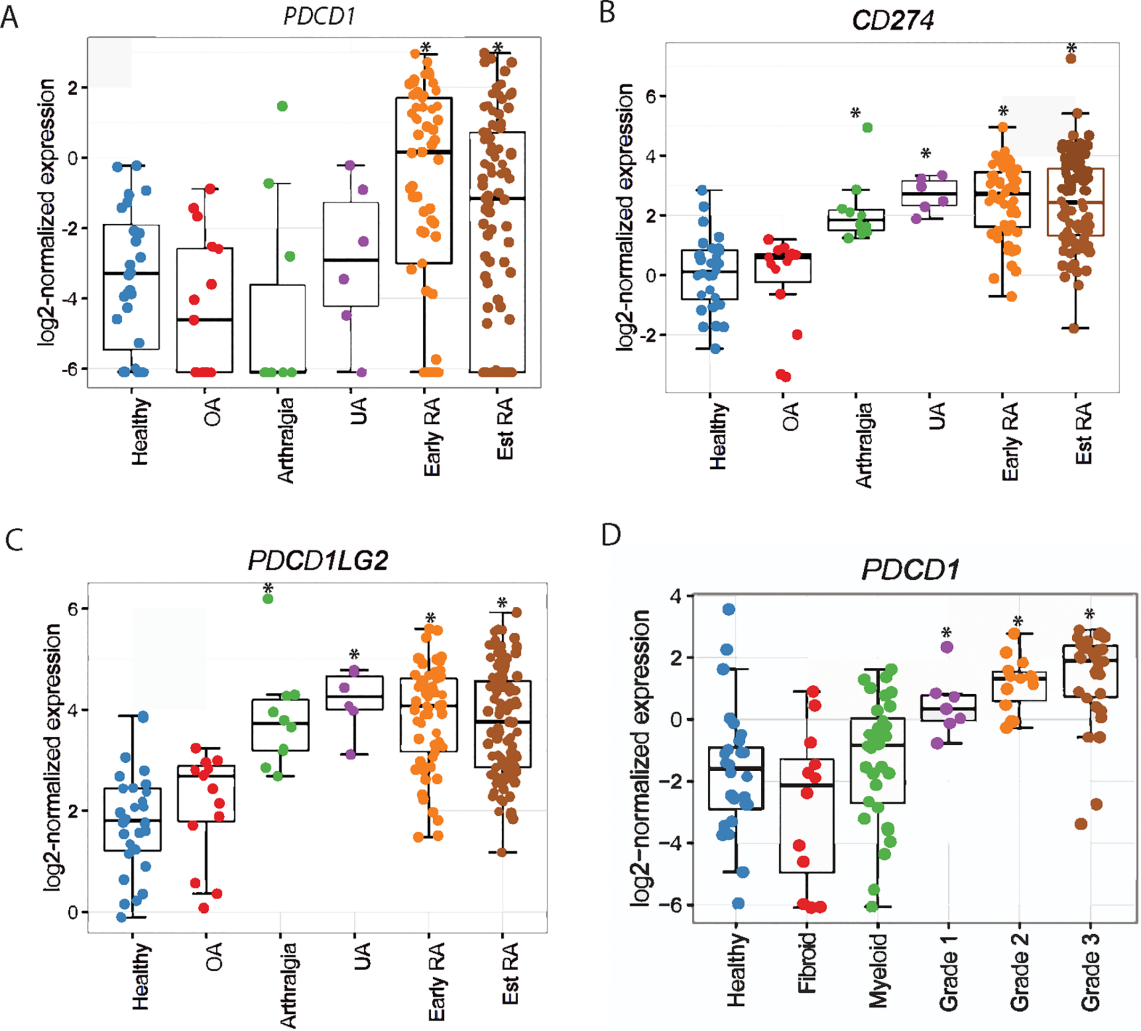
*PDCD1*, *CD274* and *PDCD1LG2* expression are increased at different stages of disease progression to RA. RNA-Seq was performed on synovial biopsies from healthy tissue, OA, arthralgia, IA/UA, early RA, and established RA (Est RA) patients. *PDCD1* (A), *CD274* (B) and *PDCD1LG2* (C) mRNA expression in synovial biopsies of different groups is shown. D. *PDCD1* mRNA in synovial tissues of three different RA disease pathotypes (fibroid, myeloid and lymphoid). Grade 1–3 indicates the severity of the disease in lymphoid pathotype. In all plots, * indicates statistically significant difference compared to healthy tissue (adjusted P<0.05).

The development of IA and synovitis in some of the cancer patients treated with Nivolumab or Pembrolizumab prompted us to examine if PD-1-associated pathway plays any role in driving disease progression and if it could be therapeutically exploited for the prevention and treatment of RA. The involvement of PD-1 pathway in RA disease progression was evaluated by obtaining synovial tissue biopsies from patients with arthralgia, IA/UA, early and established RA. Transcriptomics analysis by RNA-Seq on synovial tissue biopsies from healthy donors (n = 28), osteoarthritis (OA, n = 22), ACPA+ve RF+ve arthralgia (n = 10), IA/UA (n = 6), early RA (n = 57), and established RA (n = 95) patients revealed that *PDCD1* (encoded PD-1), *CD274* (encoded PD-L1) and *PDCD1LG2* (encoded PD-L2) expression was not increased in OA compared to healthy controls (Fig 1A). However, increased *PDCD1* expression was observed in early RA (fold change (FC) = 2.52, P = 4.38E-04) and established RA (FC = 1.49, P = 2.63E-02) synovial tissue samples, but not in arthralgia or IA/UA (Fig 1A). Interestingly, significantly higher *CD274* mRNA expression was observed even at arthralgia stage when compared to healthy control synovium samples (FC = 2.09, P = 9.50E-5; Fig 1A). CD274 expression levels were also increased in synovial tissue samples obtained from other stages of RA disease progression, namely UA (FC = 2.63, P = 1.02E-04), early RA (FC = 2.46, P = 1.07E-12) and established RA (FC = 2.39, P = 9.09E-14) (Fig 1B and Table 1). Similarly, *PDCD1LG2* was also expressed at significantly higher levels in arthralgia, IA/UA, early RA and established RA (Fig 1C and Table 1).

**Table 1:** Fold changes (log2) and adjusted P-values of *PDCD1*, *CD274* (*PD-L1*) and *PDCD1LG2* (*PD-L2*) in different group comparisons as indicated in each row. The same synovial biopsies in each group as in Fig 1 was used in the analysis.

mRNA	Group 1	Group2	Log2 Fold change	adjusted P value
*PDCD1*	normal	OA	-0.85	7.97E-01
*PDCD1*	normal	Arthralgia	-1.05	4.17E-01
*PDCD1*	normal	UA	0.49	7.90E-01
*PDCD1*	normal	Early RA	2.52	4.38E-04
*PDCD1*	normal	Est RA	1.49	2.63E-02
*PD-L1*(*CD274*)	normal	OA	-0.21	8.68E-01
*PD-L1*(*CD274*)	normal	Arthralgia	2.09	9.50E-05
*PD-L1*(*CD274*)	normal	UA	2.63	1.02E-04
*PD-L1*(*CD274*)	normal	Early RA	2.46	1.07E-12
*PD-L1*(*CD274*)	normal	Est RA	2.39	9.09E-14
*PD-L2* (*PDCD1LG2*)	normal	OA	0.36	6.47E-01
*PD-L2* (*PDCD1LG2*)	normal	Arthralgia	2.02	9.74E-07
*PD-L2* (*PDCD1LG2*)	normal	UA	2.39	4.86E-06
*PD-L2* (*PDCD1LG2*)	normal	Early RA	2.08	7.67E-15
*PD-L2* (*PDCD1LG2*)	normal	Est RA	1.94	3.90E-15

Based upon immunohistochemistry for immune cell infiltrate in the synovium, RA can be stratified into three major pathotypes, namely lymphoid (rich in B and T lymphocytes), myeloid/diffuse (infiltrate rich in monocyte/macrophages) and pausi-immune/fibroid (uninflamed and rich in fibroblasts) [20]. Increased synovial *PDCD1* expression was observed only in lymphoid but not in myeloid or fibroid pathotypes when compared to healthy synovium (Fig 1D). In addition, there was a trend towards increase in *PDCD1* expression with increased inflammation grade (1–3) associated with the lymphoid pathotype (Fig 1D). These results indicate that the synovial lymphocytes express PD-1, and it could be a biomarker for patient stratification for a potential PD-1 pathway based therapeutic.

### Majority of synovial CD4^+^ and CD8^+^ T cells show surface expression of PD-1

**Fig 2:**
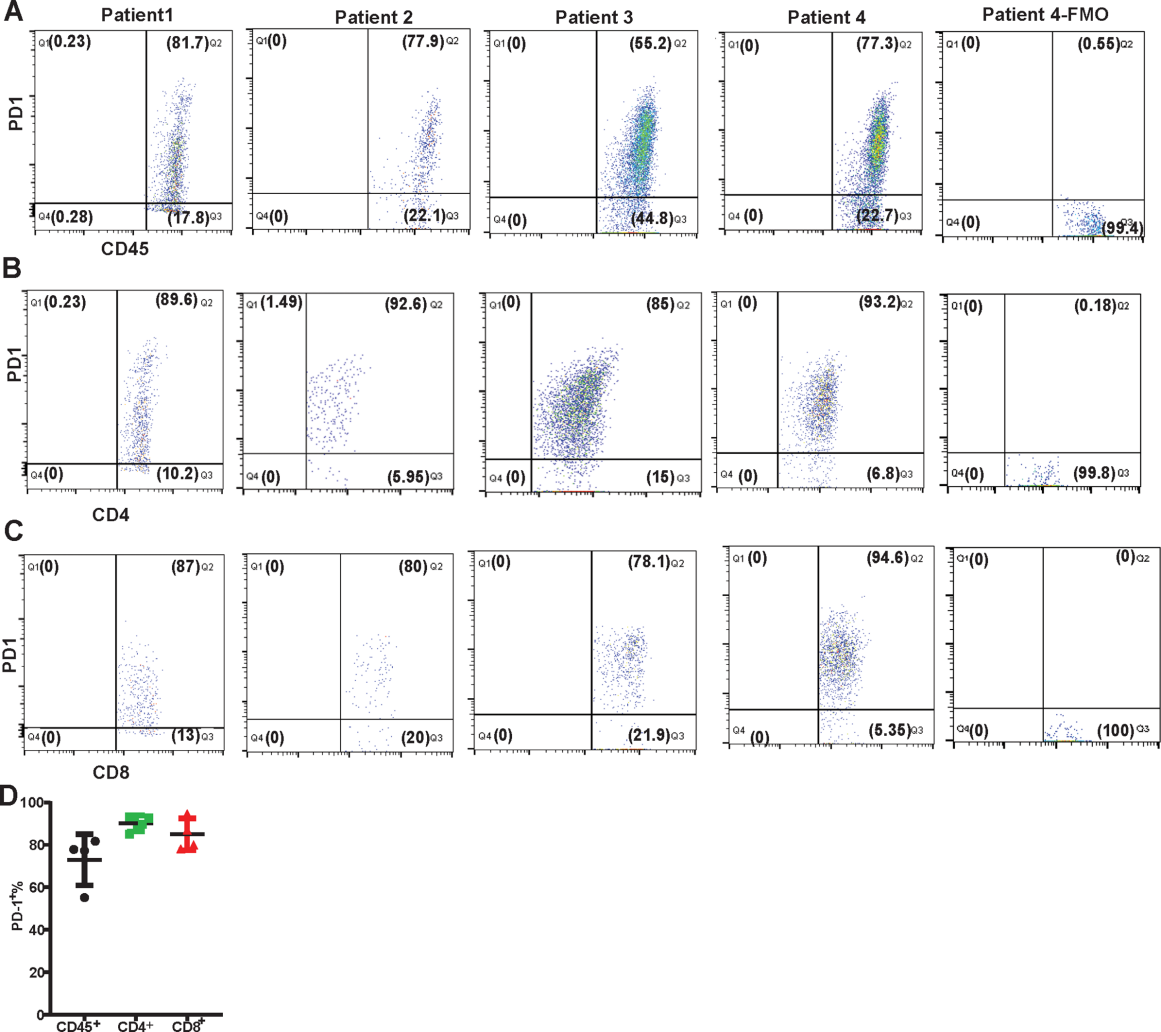
Flow cytometry analysis of surface PD1 expression on synovial tissue infiltrating immune cells. FACS plot of PD1 expression on gated live CD45^+^ lymphocytes (A), CD4^+^ (B) and CD8^+^ (C) is shown. Each FACS plot represents one individual patient. Data shown is from 4 independent patients, and pre-gated on live lymphocytes. The PD-1 fluorescence minus one (FMO) staining control for patient 4 has been shown for the CD45, CD4 and CD8 panels (A-C). D. Quantification of CD45^+^, CD4^+^ and CD8^+^ synovial infiltrating immune cells expressing PD-1 on surface is presented. Data is shown as a mean percentage of cells from 4 patients.

To evaluate surface expression of PD-1 on synovial T cells, we have isolated RA synovial tissue infiltrating immune cells and performed flow cytometry analysis. Results showed that a majority (55.2–87.1%, n = 4) of RA synovial tissue infiltrating immune cells expressed high PD-1 on their surface (Fig 2A). Further immune phenotyping revealed that most of the synovial-infiltrating CD4^+^ (85–93.2%, n = 4) and CD8^+^ (78.1–94.6%, n = 4) T cells were positive for surface PD-1 (Fig 2B and 2C). Combined data from the 4 RA patients showed that more than 82% of the synovium infiltrating CD4^+^ and CD8^+^ T cells were positive for PD-1 (Fig 2D), consistent with the presence of increased *PD1* mRNA in early and established RA synovial tissue biopsies (Fig 1A).

### Nivolumab human tumor gene signature is highly enriched in pre-RA and RA synovial tissue

**Fig 3:**
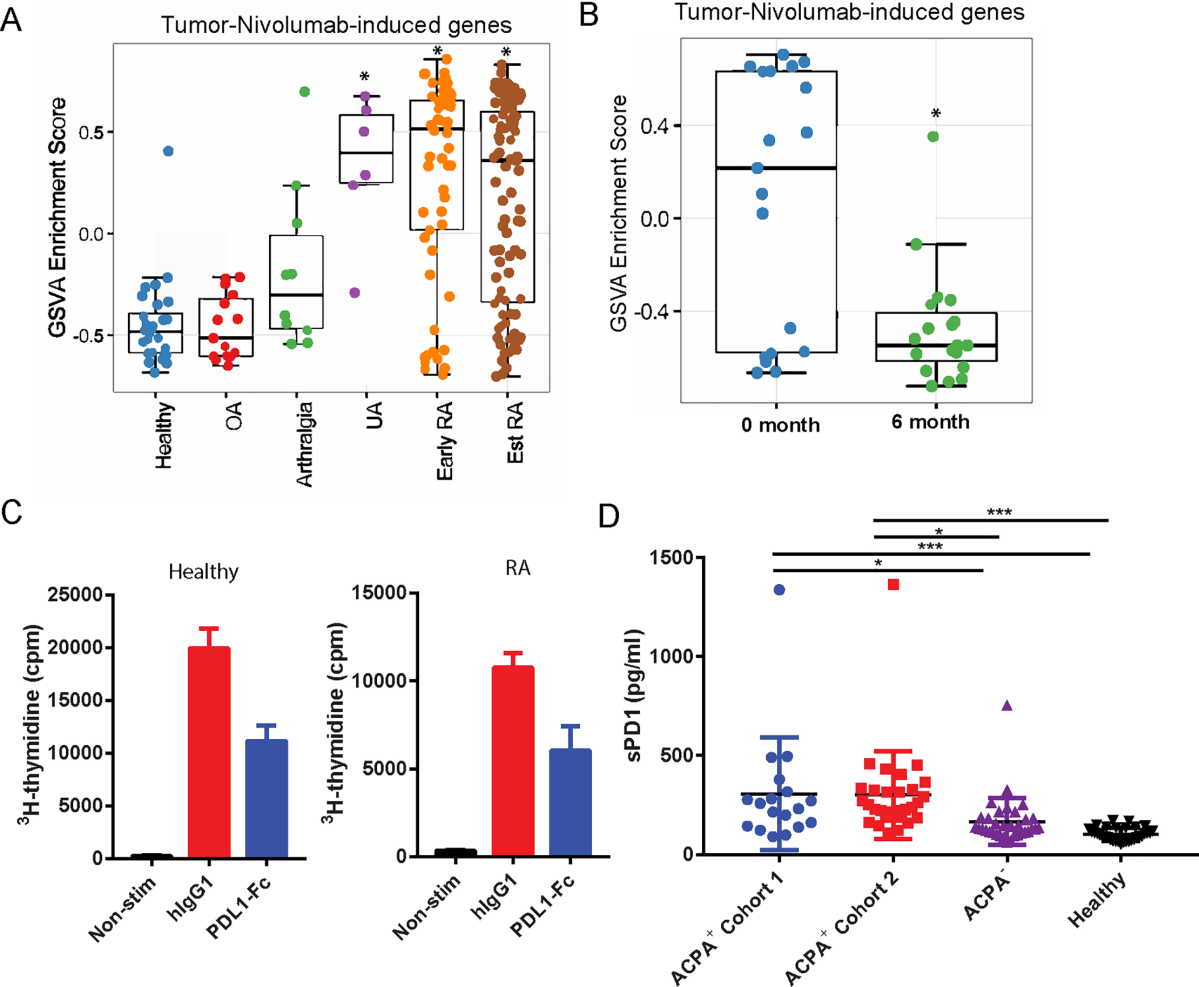
PD1 pathway is downregulated during RA disease progression. A Nivolumab signature gene set containing ~100 genes induced by the anti-PD-1 therapeutic in TME of cancer patients was used for GSVA in synovial tissue biopsy samples. A. Gene enrichment of Nivolumab up-regulated genes in healthy, OA, arthralgia, IA/UA, early RA and established RA synovial biopsies is presented. * indicates statistically significant difference compared to healthy tissue (P value <0.05). B. Nivolumab gene enrichment is reduced after tDMARD treatment. GSVA enrichment of Nivolumab gene signature pre- and post tDMARD treatment has been presented. *indicates statistically significant difference compared to pre-treatment (P<0.05). C. ^3^H incorporation by CD4^+^ memory T cells in the presence of PD-L1-Fc upon anti-CD3 activation. Data shown is representative of 3 healthy (left) and 5 RA donors (right), respectively. Error bar indicates mean±S.D. in triplicate wells. D. Soluble PD-1 levels are elevated in serum samples of ACPA+ve early RA patients. Quantification of sPD-1 in serum samples of ACPA^+^ early RA cohort 1, ACPA^+^ early RA cohort 2, ACPA^-^ early RA and healthy controls by MSD assay is presented. In D, (*) and (***) indicate statistically significant differences at P<0.05 and P<0.001, respectively.

Previous studies have shown that the treatment of cancer patients with antagonistic anti-PD1 therapeutic antibodies led to the development of arthritis and other autoimmune diseases [7–9]. The development of T cell-mediated irAE is related to the mechanism of therapeutic efficacy of the drug since it involves the activation of tumor infiltrating exhausted T cells. Choueiri et al. identified a signature of approximately 100 Nivolumab-induced genes including 71 immune lineage-associated genes, which represent the pharmacodynamic effect of the drug in metastatic renal cell carcinoma patients [19]. Given the higher expression of *PDCD1*, *CD274* and *PDCD1LG2* in pre-RA and RA synovial biopsies, and the development of IA in cancer patients, we reasoned that PD-1 pathway may be down-modulated in RA patients. Such an effect may be similar to the immune activation effect of Nivolumab in cancer patients who developed IA and other T cell-dependent autoimmune diseases. To demonstrate molecular parallelism between RA natural history of disease and anti-PD-1 antibody-induced irAE, and to evaluate the status of PD-1 signaling in synovial tissue during the development of RA, the enrichment of Nivolumab upregulated gene signature [19] was interrogated in pre-RA and RA synovial biopsies by gene set variation analysis (GSVA). Strikingly, GSVA showed that Nivolumab-induced genes in cancer patients were significantly enriched in IA/UA (P = 0.05), early (P = 1.26E-13) and established RA (P = 2.54E-14) synovial tissue samples (Fig 3A). A similar trend was also observed in arthralgia synovial tissue biopsies, which was not statistically significant possibly due to the low number of subjects. Therefore, our data demonstrate that Nivolumab-responsive genes are enriched in the synovium during RA disease progression, indicating that the PD-1 pathway is down-modulated during RA disease development. We also examined the effect of triple DMARD (tDMARD: methotrexate, sulfasalazine and hydroxychloroquine) treatment on enrichment of Nivolumab gene signature in early RA patients. Interestingly, the enrichment of Nivolumab gene signature at baseline in early RA synovial tissue samples was reduced after tDMARD treatment (P = 6.78E-04), indicating tDMARD normalized expression of those genes that represent the inhibition of PD1 pathway (Fig 3B).

We next wanted to determine if PD-1 pathway still responds to PD-L1 in RA, and therefore, could be therapeutically manipulated to down-regulate TCR-mediated signaling. Treatment of anti-CD3-activated peripheral RA memory CD4^+^ T cells with recombinant PD-L1-Fc reduced proliferation at the same level as healthy donor CD4^+^ T cells, demonstrating that even though the PD-1 pathway is down-modulated in RA, the downstream components of the pathway are intact and responsive to signaling via its natural ligand (Fig 3C).

### Serum soluble PD-1 levels are differentially upregulated in ACPA positive and ACPA negative RA

Increased serum sPD-1 levels have been described previously in established RA [21–23], and sPD-1 enhances pro-inflammatory cytokine expression and aggravates joint pathology in a murine collagen-induced arthritis model [24]. To explain the down-modulation of PD-1 pathway in RA pathogenesis and disease progression, we examined the possibility that elevated serum sPD-1 levels might interfere with the normal PD1-PDL1 signaling by sheer competition for the cell surface PD-L1/PD-L2. Therefore, we examined serum sPD-1 levels in treatment-naïve early RA cohorts. ELISA assay revealed higher sPD-1 levels in two different cohorts of ACPA +ve but not in a cohort of ACPA and RF -ve early RA patients when compared to matched healthy control serum samples (Fig 3D). These results demonstrate that sPD-1 levels are increased in ACPA+ve but not ACPA-ve early RA and in addition, imply that serum sPD-1 may participate in disease pathogenesis.

### PD-L1 protein is not available for interaction with PD-1 in RA synovial tissue

**Fig 4:**
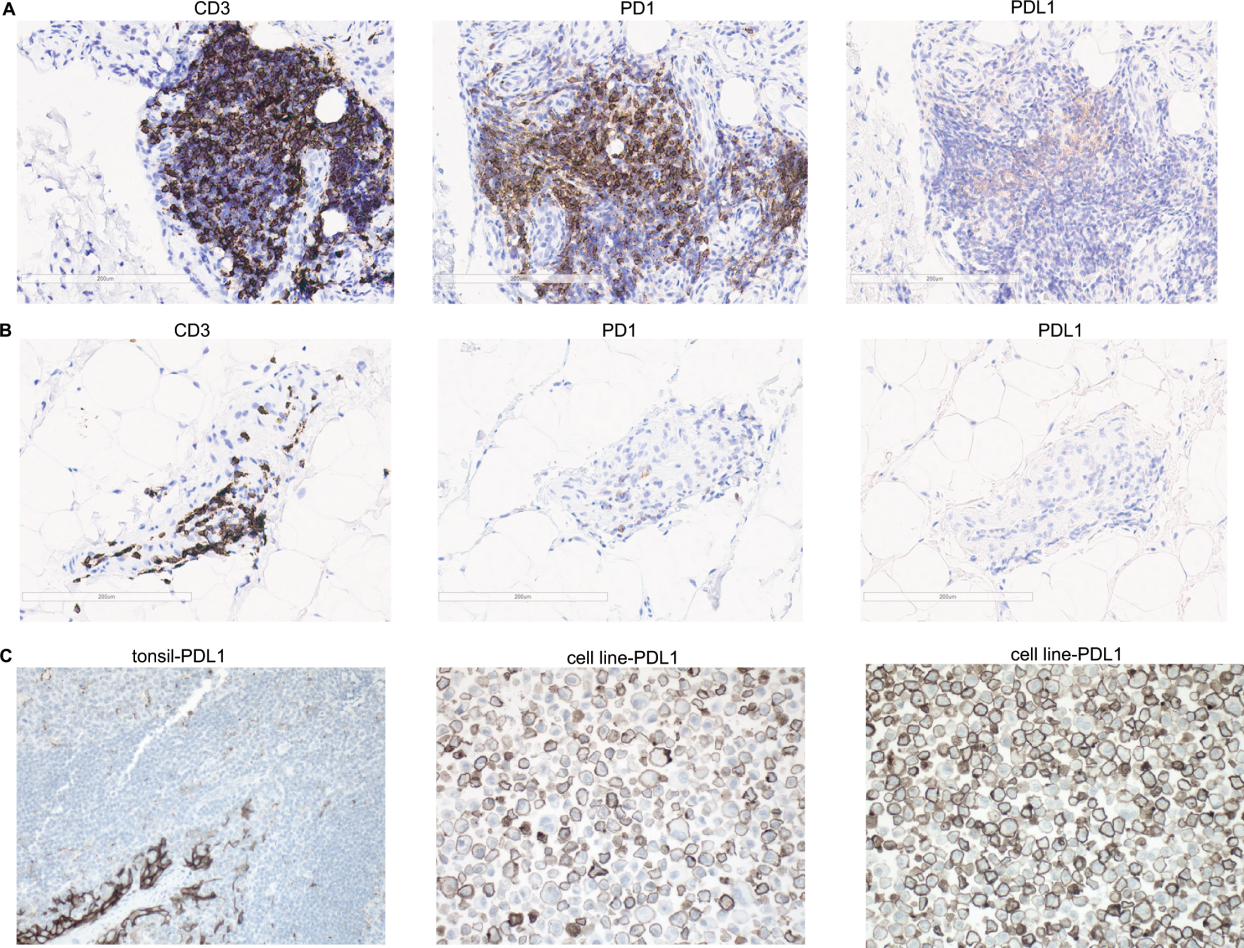
Immunohistochemistry analysis of CD3, PD1 and PD-L1 in treatment-naïve early RA synovial biopsies. A. Representative IHC of tissue sections with abundant CD3, PD1 and 5% PD-L1 staining. B. Representative IHC of tissue sections with abundant CD3 and PD1 staining but <1% PD-L1 staining. C. Positive PD-L1 staining control in human tonsil tissue (left) and positive PD-L1 staining control in cell line overexpressing PD-L1 at low (middle) and high density (right), respectively. All images are shown at 20x. Data shown in A-B represents synovial biopsies from 17 early RA patients as illustrated in Table 2.

To further evaluate the status of the PD-1 pathway during RA development, we next examined the protein expression of both PD-1 and PD-L1 in paraffin-fixed early RA synovial tissue biopsies by immunohistochemistry using an FDA-approved immunodiagnostic assay, which is routinely used for anti-PD-1 treatment decisions in clinical studies. Out of 17 early RA synovial tissue sections only 4 showed weak positive PD-L1 staining in >1% of infiltrating immune cells (Fig 4A and Table 2). Two patients (#3 and #17) showed faint PD-L1 staining in 4–5% of the infiltrating immune cells, whereas, other two (#11 and #16) showed focal weak staining in apparent lymphocytes and macrophages in a single section of the synovial tissue. Therefore, 13 early RA synovium samples were negative (either no staining or <1% of the infiltrating immune cells were weakly stained) for PD-L1 staining (Fig 4B and Table 2). In contrast, immunostaining for CD3+ T cells was readily observed in all biopsies and 2–60% of the infiltrating immune cells were positive for CD3 (Fig 4A and 4B; Table 2). PD1+ cells were observed in 15 out of 17 biopsies, and in these positive samples, 1–30% of the infiltrating immune cells were strongly positive for PD-1 (Fig 4A and 4B; Table 2). In contrast, robust PD-L1 staining was observed in the cultured PD-L1 overexpressing cells (5 cell lines) as well as in human tonsil (n = 5) tissue sections (Fig 4C). These data further support our findings that PD-1 agonist pathway is down-regulated in the RA synovial tissue since PD-L1 is not available for interaction with synovial expressed PD-1 in early RA.

**Table 2:** Quantification of PD-L1, PD1 and CD3 staining in treatment-naïve early RA synovial tissue biopsies. Table shows PD-L1^+^ at different degree, including <1%, ≥1% or ≥50%; The percentage of CD3+ and PD1+ cells as a proportion of total synovial infiltrating immune cells for each section is also presented.

Subject ID	PD-L1 Negative (<%1) (Y/N)	PD-L1 Positive (≥%1) (Y/N)	High PD-L1 Positive (≥%50) (Y/N)	CD3+%	PD1+%
1	Yes	No	No	5	0
2	Yes	No	No	10	3
3	No	Yes—4%	No	60	30
4	Yes	No	No	5	1
5	Yes	No	No	6	2
6	Yes	No	No	15	3
7	Yes	No	No	20	10
8	Yes	No	No	4	1
9	Yes	No	No	2	0
10	Yes	No	No	2	1
11	No	Yes	No	20	10
12	Yes	No	No	20	10
13	Yes	No	No	6	3
14	Yes	No	No	30	10
15	Yes	No	No	30	10
16	No	Yes	No	40	20
17	No	Yes—5%	No	30	20

## Discussion

Herein, by utilizing gene signatures derived from human metastatic renal carcinoma patients following an antagonistic anti-PD-1 antibody treatment [19], we have identified PD-1 pathway as a common molecular mechanism linking Nivolumab-mediated IA and the naturally occurring disease during RA progression. The major findings are: 1) the expression of *PDCD1*, *CD274* and *PDCD1LG2* is elevated in synovial tissues at various stages of RA disease progression; 2) PD-1 is expressed on a majority of synovial tissue infiltrating CD4+ and CD8+ T cells; 3) Nivolumab-responsive genes in TME are enriched in synovial biopsies; 4) serum sPD1 is elevated in ACPA^+^ early RA patients and, 5) PD-L1 protein is not available in the early RA synovium for agonistic interaction with PD-1.

Elevated surface expression of PD-1 on synovial fluid T cells has been documented [21, 25–28]. However, the expression of PD-1/PD-L1/PD-L2 during RA disease progression and the activity status of the PD-1 pathway in RA at various stages of disease development has not been reported. Herein, we have demonstrated that the synovial tissue expression of *PDCD1* was upregulated in early and established RA, and that of its ligands (*PD-L1* and *PD-L2*) was significantly induced in arthralgia, IA/UA, early and established RA in comparison to their OA and healthy control counterparts (Fig 1A–1C). Importantly, we demonstrated that 55–80% of the synovial tissue infiltrating cells (Fig 2A) and more than 80% of the RA synovial tissue infiltrating CD4^+^ and CD8^+^ T cells were PD-1^+^ (Fig 2B–2D). These observations were unexpected and counterintuitive since PD-1 serves as a checkpoint inhibitory molecule to tone down TCR-mediated responses and T cell autoimmunity [5, 29], and we have observed an increase in the expression of PD-1 in the disease tissue with rampant T cell activation. Studies in PD-1 deficient mice demonstrated that disruption of PD-1 results in the breakdown of tolerance and development of autoimmunity, such as lupus, arthritis and type I diabetes [30–32]. One would have expected that T cell-mediated autoimmunity might involve aberrant expression of co-signaling receptors, with increased expression of co-stimulatory (e.g., CD28) and downregulation of co-inhibitory checkpoint (e.g., PD-1 and CTLA-4) molecules in autoimmunity. As expected, in human tumors there was a markedly increased surface expression of co-inhibitory molecules, including PD-1, on tumor infiltrating lymphocytes [33]. The clinical success of PD1-PDL1 pathway blockade proves PD1 pathway is functionally inhibitory in TME. Not unexpectedly, these checkpoint therapeutics have resulted in T cell-mediated irAE including colitis, arthralgia and inflammatory arthritis [7–9]. In view of the observations that immune checkpoint inhibitors induce T cell-mediated autoimmune symptoms, our results described herein provide an underlying basis of the joint involvement in cancer patients. The molecular parallelism between the natural history of RA disease progression and Nivolumab or anti-PD-1 antagonist gene signature suggests that the PD-1 co-inhibitory pathway dysregulation may result in breakdown of immune tolerance in autoimmunity development in both cancer and RA patients. Since PD-1 expression was observed in both tumor infiltrating T cells [33] and synovial tissue infiltrating T cells (Figs 1 and 2), we reasoned that unlike the functional PD-1 pathway in TME, the same pathway is dysfunctional in RA. That is, even though most of synovial tissue infiltrating CD4^+^ and CD8^+^ T cells are PD-1^+^ (Fig 2B–2D), the disease still progresses from an early stage to established RA.

Nivolumab, an antagonistic PD-1 antibody, gene signature has been reported from tumors of renal cell carcinoma patients [19]. We hypothesized that if the PD-1 pathway activity is dysregulated in RA, then we may observe an enrichment of Nivolumab-regulated genes in synovial biopsies. Interestingly, a statistically significant enrichment of TME Nivolumab gene signature was observed not only in established RA but also in IA/UA and early RA, and in addition, there was a trend towards increased enrichment even in arthralgia synovial tissue, albeit not statistically significant (Fig 3A). These results demonstrated that PD-1 pathway was down-modulated in established RA as well as during the natural evolution of the disease from IA/UA to RA. The induced expression of a common set of genes between Nivolumab-treated TME and synovial tissue biopsies demonstrates that the PD-1 pathway-responsive T cells are unrestrained in RA in a manner analogous to anti-PD1 antagonistic antibody treatment of cancer patients. Therefore, restoring the inhibitory activity of the PD-1 pathway through an agonistic anti-PD-1 antibody may restrain autoimmune T cells in pre-RA and RA. The enrichment of Nivolumab gene signature in pre-RA and RA synovial biopsies may not be specific to the PD-1 pathway and they could be upregulated by other PD-1 non-specific mechanisms. Since Nivolumab is a PD-1 antagonist, the enrichment of its gene signature logically indicates that the Nivolumab pathway is enriched during RA disease progression, or in other words PD-1 pathway antagonism is observed at various stages of RA disease development. Since PD-1 antagonism pathway genes are in fact involved in T cell activation, which accounts for the therapeutic efficacy of Nivolumab in cancer patients, it is not far-fetched to predict that similar set of T cell genes may show induction after Nivolumab treatment and in autoimmune settings such as RA. Our results provide experimental evidence to this hypothesis. Although we cannot rule out the possibility that the Nivolumab gene signature is upregulated in RA due to dysregulation of other T cell pathway(s), our results still indicate that upregulated genes are PD1-responsive and therefore, the gene signature and the activity of T cells could be potentially down-modulated with a PD-1 pathway agonist therapeutic. The down-modulation of the PD-1 pathway is not because of inherent defective signaling in RA, since exogenous PD-L1-Fc was able to transduce its inhibitory signal in CD3-activated CD4^+^ T memory cells from RA patients, leading to decreased cell proliferation (Fig 3C). These results agree with Raptopoulou et al., (26) who have reported inhibition of proliferation of anti-CD3-stimulated peripheral blood and synovial fluid CD4+ T cells by human PD-L1-Fc (26). Similarly, Wan et al., (21) have also reported that RA synovial CD4+ T cells are susceptible to human PD-L1-Fc-mediated inhibition of cell proliferation. However, Bommarito et al., (23) have reported that in contrast to healthy donors, RA periphery and synovial fluid CD4+ T cells do not respond to PD-L1-Fc-mediated inhibition of proliferation. Differences in ACPA/RF status and/or the use of DMARDs/biologics in various study RA cohorts could result in divergent outcomes.

The in vitro and in vivo proinflammatory properties of sPD-1 have been well documented in the literature. sPD-1 has been reported to enhance T- cell responses by interfering with PD-1/PD-L1 pathway in autoimmune, tumor and viral infection systems [34–39]. Furthermore, since sPD-1 aggravates progression of symptoms in a murine model of collagen-induced arthritis [24], and increased serum sPD-1 has been reported in RA patient [21, 22, 24], we examined the possibility that increased sPD-1 may interfere with cell surface PD-L1 and T cell-bound PD-1 resulting in defective PD-1 pathway signaling. In accordance with previous observations in established RA, we show that sPD-1 levels were increased in early RA compared to healthy control serum samples (Fig 3D). Importantly, we discovered that sPD-1 levels were increased significantly only in ACPA positive but not in ACPA negative early RA (Fig 3D). Although some of the ACPA-ve subjects showed increased sPD-1 levels, however overall as a group ACPA-ve subjects did not show any statistically significant difference when compared to healthy controls. It could be because some of the RA subjects classified as ACPA-ve in the CCP assay may show positivity to some of the ACPA sub-specificities. We did not see any significant association of sPD-1 levels with DAS28 or ACPA levels. Since another mechanism of down-regulation of PD-1 pathway in synovium could be decreased expression of PD-L1, we examined synovial PD-L1 protein levels by immunohistochemistry. The results revealed that although PD-L1 protein was readily detectable in tonsil tissue sections and PD-L1 overexpressing cells lines, its expression was not observed in most of the early RA synovial tissue biopsies, thus indicating that in the synovial tissue, PD-L1 protein is not available for agonistic interaction with the widely expressed PD-1 (Fig 4 and Table 2). In contrast, most of the synovial tissue biopsies showed the presence of CD3 and PD-1 proteins (Table 2). Our immunohistochemistry results differ from a published report, where the authors have shown positive PD-L1 staining in the OA and RA synovial tissue sections (26), which could be because of the use of different antibodies. We have used IHC 22C3 pharmDx, a FDA-approved clinical diagnostic test for PD-L1 immunostaining, which utilizes a very well-validated anti-PD-L1 antibody. Taken together our observations demonstrate that despite increased expression of PD-1 in RA synovial tissue infiltrating T cells, the PD-1 pathway is down-regulated at various stages of RA disease progression plausibly through increased serum sPD-1 and decreased synovial PD-L1 protein expression. Thus, modulating the pathway by agonistic PD1 therapeutic antibodies may not only show efficacy in the treatment of RA but also in disease interception by preventing the progression to RA.
